# Treacher Collins Syndrome: A Case Report and a Brief Review on Diagnostic Aids

**DOI:** 10.5005/jp-journals-10005-1116

**Published:** 2011-04-15

**Authors:** Sowmya B Shetty, Ann Thomas, Raghavendra Pidamale

**Affiliations:** 1 Professor and Head, Department of Pedodontics and Preventive Dentistry, AJ Institute of Dental Sciences, Karnataka, India; 2 Professor, Department of Pedodontics and Preventive Dentistry, AJ Institute of Dental Sciences, Karnataka, India; 3 Assistant Professor, Department of Pedodontics and Preventive Dentistry, AJ Institute of Dental Sciences, Karnataka, India

**Keywords:** Treacher Collins syndrome, Mandibulofacial dysostosis, Treacle, Clinical features, Treatment.

## Abstract

Treacher Collins syndrome (Mandibulofacial dysostosis) is characterized by deafness, hypoplasia of facial bones (mandible, maxilla and cheek bone), antimongoloid slant of palpebral fissures, coloboma of the lower lid and bilateral anomalies of the auricle. Hypoplasia of the facial bones may be the first indicator of the disorder. We present a case report of Treacher Collins syndrome with their extraoral findings, intraoral findings and their treatment plan. We have also included the various etiological factors, clinical diagnostic aids, and multidisciplinary team approach.

## INTRODUCTION

Treacher Collins syndrome (TCS), also called Treacher Collins-Franceschetti syndrome or mandibulofacial dysostosis, is an autosomal dominant disorder affecting the development of structures derived from the first and second brachial arches during early embryonic development.^1^ The estimated incidence of TCS ranges from 1:40,000 to 1:70,000 of live births. TCS is characterized by deafness, hypoplasia of facial bones (mandible, maxilla and cheek bone), antimongoloid slant of palpebral fissures, coloboma of the lower lid and bilateral anomalies of auricle. It is a condition in which the cheek bones and jawbones are underdeveloped.^2^

**Fig. 1:**
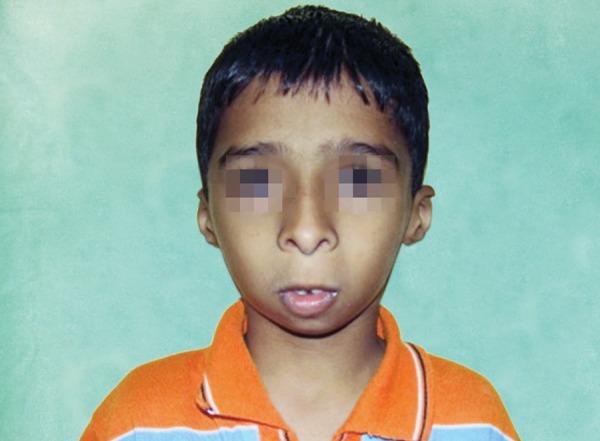
Frontal view of the patient: Clinical signs

## CASE REPORT

A 9-year-old boy reported to the department of pedodontics and preventive dentistry, AJ institute of dental sciences, Kuntikana, Mangalore; complaining of retained deciduous teeth in the upper anterior region. The patient’s mother also complained of a symmetry of his face and deafness (Fig. 1).

**Fig. 2:**
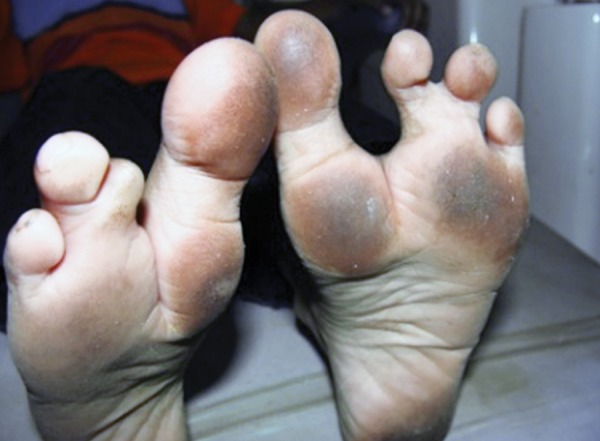
Webbing of toes

On general examination, the patient was of short stature (115 cm), low weight (18 kg) and pallor was observed. On both the lower limbs, the 2nd toe was missing and webbing of the 3rd and 4th toes was seen (Fig. 2).

### Extraoral Findings

*Ophthalmologicalfindings:* Bilateral corneal opacity with adherent leucoma. On examination, the fundus was bilaterally hypermetropic with macular dystrophy.

**Fig. 3:**
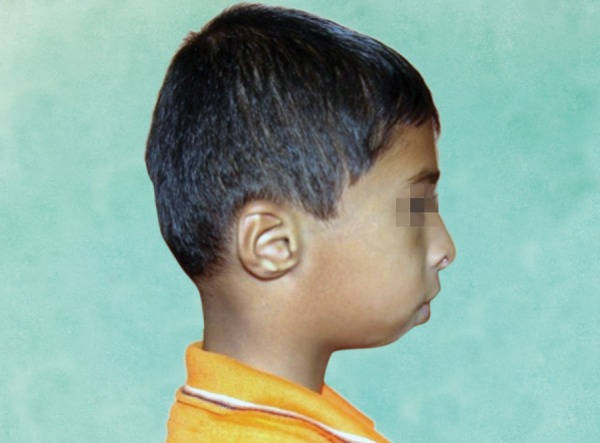
Lateral view of the patient (profile): Clinical signs

*ENT findings:* Conductive hearing loss, large and everted ears, narrow nasal cavity, soft palate drooping downward (Fig. 3).

*Facial dysmorphic features:* Microcephaly, micrognathia, hypoplastic mandible.

### Intraoral Findings

High-arched palate, malocclusion, mouth breathing, V-shaped lower arch, open bite, micrognathia, microstomia, incompetent lips, retained deciduous anterior teeth, caries in relation to 16, 36, 46 root stumps in relation to 54, 64, 65, 85 and preshedding mobility in relation to 71, 81.

**Fig. 4:**
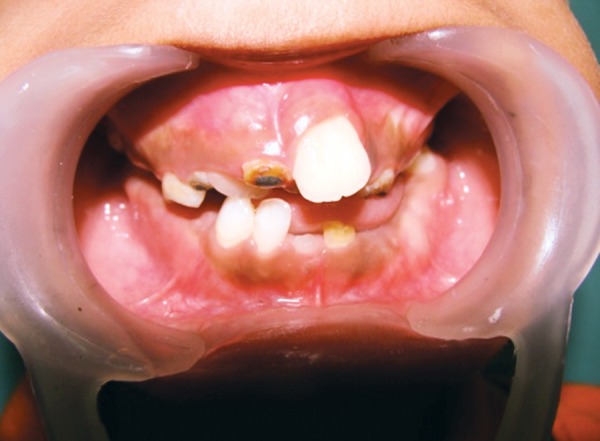
Intraoral photograph showing retained deciduous teeth, missing permanent teeth and anterior crowding

**Fig. 5:**
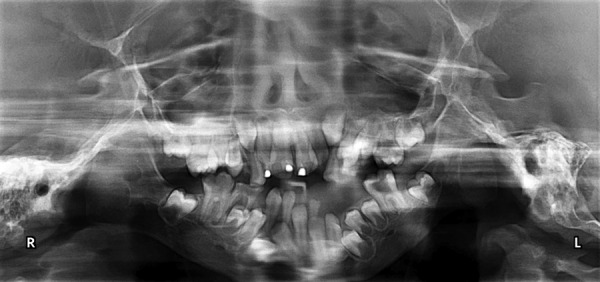
Orthopantomograph showing retained deciduous teeth, crowding, periapical lesion in relation to 46

**Fig. 6:**
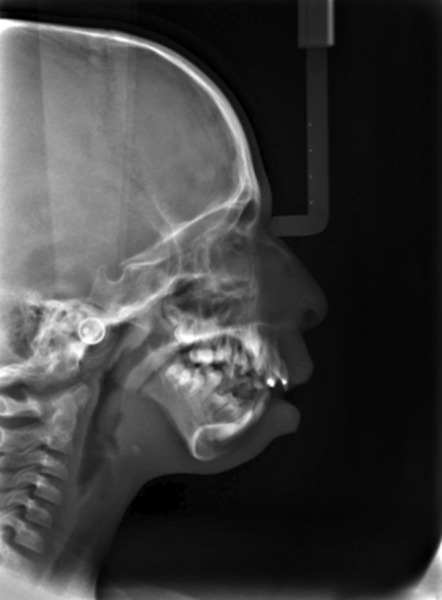
Lateral cephalogram showing prominent antegonial notch and underdeveloped zygoma

The child already had a hearing aid and he was undergoing speech and hearing therapy (Figs 4 to 6).

**Fig. 7:**
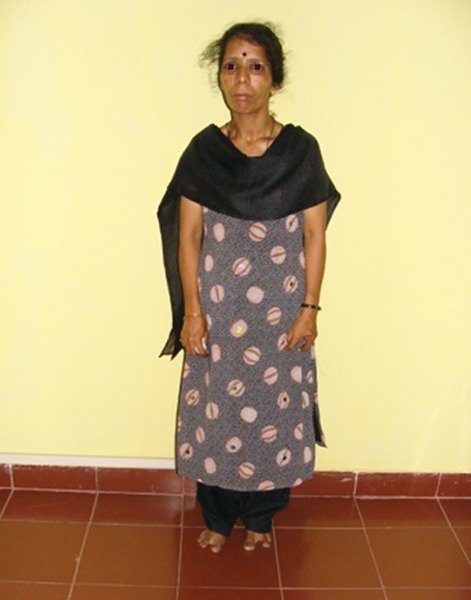
Mother of the child

**Fig. 8:**
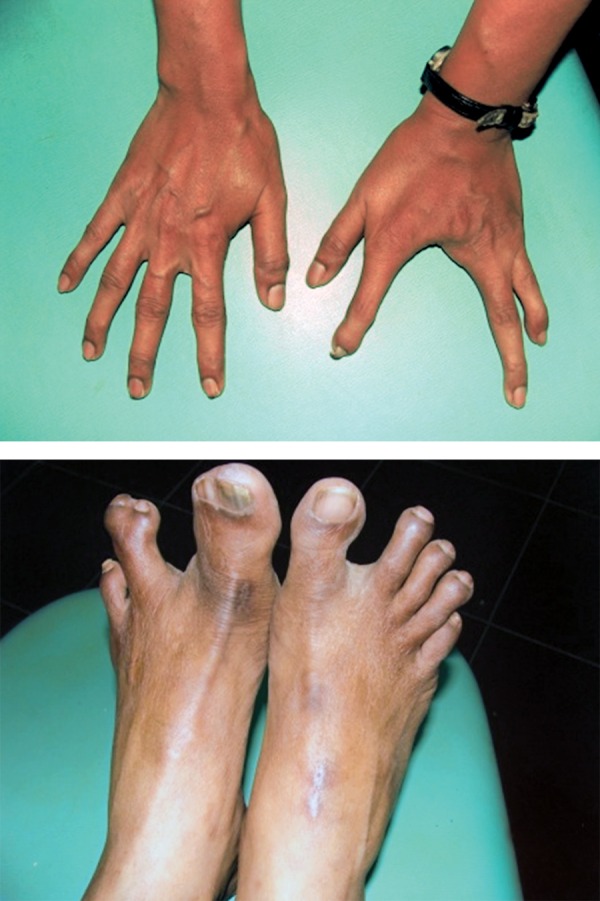
Webbing of fingers and toes of the mother

His mother also presented with similar features with lesser clinical severity (Figs 7 and 8).

### Investigations

Orthopantomograph, lateral cephalogram, CT, blood investigations.

### Diagnosis

Based on the history and records, clinical features and ra-diographic examination, he was diagnosed as having TCS.

## TREATMENT

**Fig. 9:**
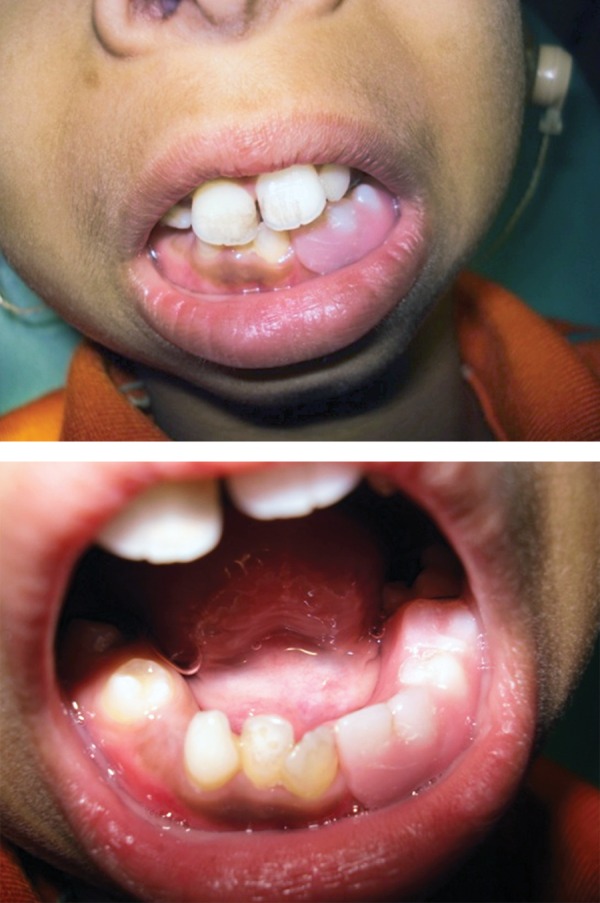
Posttreatment photograph showing corrected anterior teeth with a removable partial denture

The clinical condition demanded the need for removal of retained root stumps of deciduous teeth, restorations and root canal treatment followed by an artificial prosthesis for mastication till the eruption of the permanent teeth. Before starting the treatment we consulted a pediatrician, ENT surgeon, ophthalmologist and anesthetists. After obtaining their consent we completed our treatment. The boy was also advised by the ophthalmologists to wear spectacles daily; otherwise he would end up with amblyopia (lazy eyes) (Fig. 9).

## DISCUSSION

### History and Etiology

Thomson was the first to refer to this syndrome in 1846. In 1900, Dr E Treacher Collins, a British ophthalmologist, described two children who had very small cheek bones and notches in their lower eyelids. Therefore, the condition gets its name from him. For unaffected parents with one child with TCS, the chance of giving birth to a second child with the condition is negligible. Adults with TCS have a 50% chance of passing the condition to the offspring. When a parent with TCS passes on the genes, the children may be affected in varying degrees. The degree may be the same as the parent, milder or more severe. There are two possible ways that TCS develops. First, TCS can develop as a new mutation. This means that both parents pass on normal genes to their child. The second way that TCS develops is by inheriting it from one of the parents.^2^The only gene currently known to be associated with TCS is TCOF1 which is mapped to chromosome 5 q31.3-q33.3, encoding a serine/alanine-rich protein, called ‘treacle’.^3^ There is no preference among genders or races and it consists of autosomal dominant trait of variable expressiveness. Its phenotypical expression probably results from bilateral congenital malformation involving the first and second brachial arches.^4,5^

### Pathogenesis

Neural crest cells migrate over extensive distances to the periphery of the face giving rise to most of the cartilage, bone, connective and peripheral tissues in the head. Most disorders of craniofacial development are thought to be caused by defects in the formation, proliferation, migration and/or differentiation of cranial neural crest cells and TCS is no exception. Hence, abnormal neural crest migration, ectopic cell death and inappropriate differentiation have all been hypothesized as underlying causes of TCS. Therefore, focuses on recent advances in our understanding of the basic etiology, pathogenesis and emerging prospects for prevention are required.^6,7^

### Diagnostic Clinical Features

These are the diagnostic features of TCS:

- I. *Eyes*
  - Antimongoloid slant of the palpebral fissures
  - Colobomata and hypoplasia of the lower lids and lateral canthi
  - Partial absence of eyelid cilia
  - Hypertelorism.
- *Ears*
  - External ear anomalies
  - External auditory canal abnormalities
  - Middle ear cavity ossicular deformities
  - Conductive hearing loss results from variable degrees of hypoplasia of the external auditory canals and ossicles of the middle ears.
- *Nose/mouth*
  - Respiratory compromise in severely affected patients as a result of the following two factors:
    - Presence of maxillary hypoplasia, which tends to constrict the nasal passages and results in a degree of choanal stenosis or atresia.
    - Presence of mandibular micrognathia and a retro-positioned tongue obstructing the oropharyngeal and hypopharyngeal spaces.
  - Nasal deformity
  - Microstomia
  - Cleft palate with or without cleft lip
  - High-arched palate
  - Malocclusion
  - Open bite.
- *Facial bone malformation―the most characteristic findings are as follows:*
  - Hypoplasia of the malar bones
    - Often with clefting through the arches.
    - Limited formation of the residual zygomatic complex.
  - Orbits
    - Hypoplastic lateral aspects of the orbits
    - Dysplastic inferior lateral orbit.
  - Maxilla and mandible
    - Characteristically hypoplastic
    - Variable effects on the temporomandibular joints
    - Anterior open bite
    - A steep occlusal plane.
- Sleep apnea and sudden infant death syndrome.^1,3,8^

### Differential Diagnosis

- Nager’s acrofacial dysostosis
- Miller acrofacial dysostosis
- Oculoauriculovertebral spectrum.^1^

### Diagnostic Tests

1. Radiographs and CT for evaluation of craniofacial abnormality
2. Audiological evaluation for hearing impairment
3. *DNA diagnosis:* Direct sequencing of the coding and flanking intronic regions of TCOF1 defects mutations in about 90 to 95% of patients.^1^

### Prenatal Diagnosis for Pregnancies

- Two-dimensional and preferably three-dimensional sonography
  - Polyhydramnios
  - Demonstration of characteristic facies of TCS, like downward slanting palpebral fissures, microgna-thia, abnormal appearance of the nose with narrow nostrils, cleft lip/palate, low set dysplastic ears and abnormal fetal swallowing.
- Amniocentesis or CVS
  - To detect TCOF1
  - The disease causing allele of an affected individual must be identified before prenatal testing can be performed
  - The presence of a TCOF1 mutation detected by prenatal diagnosis does not predict the specific malformation or severity of the disease.^1^

## MANAGEMENT OF TCS

The current approach for TCS’s clinical deformities seeks functional and esthetical corrections as well as psychosocial support. Multidisciplinary approach, including otorhino-laryngologists, craniofacial surgeons, ophthalmologists, speech therapists, psychologists and pediatric dentist, is the most appropriate way to manage these patients. In addition to anatomical and physiological anomalies, TCS patients carry a social stigma because of its severe facial deformities.^9^ Depending on the severity of the TCS, the patient may need some or all of the following procedures: A conductive hearing aid, correction of the cleft palate, repair of the sidewall and floor of the eye socket, repair of cheek bones, repair of eyelid notches, correction of the undeveloped jaw and chin, surgery to correct the beak-like nose, reconstruction of the ear.^10^

The major problem in anesthesia lies on the maintenance of a free airway and intubation. During postoperative period, pharyngeal and laryngeal edema may develop even after pharyngoplasty. Cases of sleep apnea, respiratory distress and even sudden death have been reported.^4,6,7^

Since the children with TCS often have problems with their teeth and jaw, the pediatric dentist will care for these specialized problems. There are circumstances in which teeth are absent or a patient may be unable to open the mouth. This makes the care of their teeth quite difficult; therefore, the special skills of a pediatric dentist are needed.^2^

## CONCLUSION

TCS is an autosomal dominant disorder of craniofacial development which presents with unusual clinical features associated with abnormalities of structures derived from the first and second brachial arches, including antimongoloid slant of palpebral fissures, colobomas of the lower eyelid, eyelash malformations, deafness, hypoplasia of facial bones (mandible, maxilla and cheek bone) and malar and mandibular defects. Management of TCS needs a multidisciplinary approach. The treatment plan is made to meet the individual patients need, considering the growth patterns, function and psychological development.
